# Hepatic ferroptosis induced by *Clonorchis sinensis* exacerbates liver fibrosis

**DOI:** 10.1371/journal.pntd.0013164

**Published:** 2025-06-02

**Authors:** Haoyang Zhang, Xiaocen Wang, Xu Zhang, Yeting Ma, Penglin Bao, Yanhui Yu, Yuru Wang, Pengtao Gong, Nan Zhang, Soon-Ok Lee, Xin Li, Jianhua Li

**Affiliations:** 1 State Key Laboratory for Diagnosis and Treatment of Severe Zoonotic Infectious Diseases, Key Laboratory for Zoonosis Research of the Ministry of Education, Institute of Zoonosis, and College of Veterinary Medicine, Jilin University, Changchun, China; 2 Second Affiliated Hospital, Jilin University, Changchun, China; 3 College of Public Health, Jilin Medical University, Jilin, China; 4 Department of Medical Research Center for Bioreaction to Reactive Oxygen Species, Biomedical Science Institute, School of Medicine, Graduate School, Kyung Hee University, Seoul, Republic of Korea; Salk Institute for Biological Studies, UNITED STATES OF AMERICA

## Abstract

*Clonorchis sinensis* (*C. sinensis*) is a food-borne zoonotic parasite link to liver fibrosis and cholangiocarcinoma. Limited understanding of its mechanisms in causing liver fibrosis has impeded therapeutic advances for *C. sinensis*-induced liver lesions. Ferroptosis, a novel form of cell death involving iron overload and lipid peroxidation, exacerbates liver fibrosis; however, its role in *C. sinensis* infection remains unexplored. In this study, ferroptosis were detected in *C. sinensis*-infected C57BL/6 mice as well as in AML12 cells stimulated by *C. sinensis* excretory/secretory products (ESPs). 12 ferroptosis related genes were screened and we found glutathione peroxidase 4 (GPX4, 7 d), solute carrier family 7 member 11 (SLC7A11, 7 d) and nuclear factor erythroid 2 related factor 2 (Nrf2, 35 d) was significantly decreased in mice. Western blot results confirmed *C. sinensis* and ESPs down-regulated the expression of GPX4, SLC7A11 and Nrf2. GSH depletion, malondialdehyde (MDA) accumulation, mitochondrial structure damage, and iron overload were found in *C. sinensis-*infected mice and ESPs-stimulated AML12 cells, suggesting that ferroptosis occurred *in vivo* and *in vitro*. Treatment with ferroptosis inhibitor Fer-1 in *C. sinensis-*infected mice alleviated ferroptosis, reduced the productions of IFN-γ, TNF-α, IL-12 and IL-6, and downregulated transforming growth factor (TGF)-β/Smad pathway activation. In AML12 cells, Fer - 1 pretreatment reduced ESPs - induced ferroptosis and IL-6, TNF-α production. Fer - 1 also alleviated liver lesions, reduced parasite load (65%), α-SMA expression and collagen fiber deposition in infected mice. In conclusion, *C. sinensis* could cause ferroptosis, which promoted the secretions of IL-6 and TNF-α as well as the activation of TGF-β/Smad pathway, leading to exacerbated liver fibrosis.

## Author summary

*Clonorchis sinensis* is a zoonotic parasite that can induce host liver fibrosis and even cholangiocarcinoma. Further study of the pathogenic mechanism of *C. sinensis* will help us develop new therapeutic targets for clonorchiasis. Ferroptosis is a newly defined type of programmed cell death, which is closely related to the occurrence of liver fibrosis, but the role of ferroptosis in *C. sinensis*-induced liver fibrosis has not been fully elucidated. In our study, ferroptosis was measured *in vitro* and *in vivo* by *C. sinensis* infection of C57BL/6 mice and stimulation of mouse normal hepatocytes AML12 with *C. sinensis* ESPs, respectively. And the ferroptosis inhibitor Ferrostain-1 (Fer-1) was used for treatment. The treatment with Fer-1 alleviated the *C. sinensis*-induced liver ferroptosis *in vivo* and the ESPs-induced AML12 ferroptosis *in vitro*, in addition to reducing the parasite load, alleviating liver lesions, and decreasing the expression of pro-inflammatory cytokines. In addition, inhibiting ferroptosis suppressed the TGF-β/Smad2/3 pathway and liver collagen deposition in *C. sinensis*-infected mice. These results indicate that inhibiting ferroptosis can alleviate liver fibrosis caused by *C. sinensis*. Our results contribute to understanding the molecular mechanism of *C. sinensis*-induced host liver fibrosis and provide insights into developing novel therapeutic targets for this disease.

## 1. Introduction

Clonorchiasis, also known as liver fluke disease, is a zoonotic parasitic infection primarily caused by *Clonorchis sinensis* (*C. sinensis*) parasitizing the bile ducts of humans or animals, leading to hepatobiliary pathologies [1]. Liver fluke is prevalent in several countries, including China, South Korea, Vietnam and the Russian Far East, and infects more than 15 million people, of which over 85% are in China [2]. Adult worms parasitize the bile ducts and cause host gallstones, cholangitis, liver fibrosis, and even cholangiocarcinoma, thus being classified as a class I carcinogen [2,3]. Chronic liver injury caused by *C. sinensis* is mainly due to mechanical stimulation of worm migration, obstruction of bile ducts, and toxic stimulation by *C. sinensis* excretory/secretory products (ESPs), which in turn leads to the proliferation of myofibroblasts in the portal area, collagen fiber deposition, and eventually liver fibrosis. Praziquantel serves as the primary therapeutic agent for clonorchiasis, but there are risk of drug resistance and side effects such as headaches, nausea, and abdominal discomfort associated with its administration [4]. Currently, no clinically approved vaccine is available for *C. sinensis*. Given the widespread prevalence of clonorchiasis and its negative impact on public health, a comprehensive understanding of *C. sinensis* pathogenic mechanisms will help us to develop new strategies for prevention and treatment of clonorchiasis.

Patients with clonorchiasis pathologically present with hyperplasia of bile duct epithelial cells, surrounding hepatocytes atrophy and edema, and active proliferation of fibrous connective tissue, showing typical manifestations of liver fibrosis [2]. *C. sinensis* induces chronic oxidative stress in the host liver which is considered as an important mechanism contributing to the development of liver fibrosis [5]. The excessive reactive oxygen species (ROS) generated during this process results in molecular damage and organelle dysfunction. Notably, the excessive production of lipid ROS may trigger ferroptosis, a distinct form of programmed cell death.

Recent research has demonstrated that ferroptosis is one of major contributors to liver fibrosis and presents a viable therapeutic target for its treatment [6]. Ferroptosis, a form of iron-dependent programmed cell death, was initially proposed by Dixon’s team in 2012. This process is primarily characterized by an excessive accumulation of iron and lipid peroxidation [7]. When the body experiences an excessive accumulation of iron and lipid peroxidation, these conditions can significantly overwhelm the antioxidant defense systems. Consequently, lipid peroxides begin to accumulate on cellular membranes, leading to their rupture and triggering a process known as ferroptosis [8]. The ferroptosis process primarily involves the reduction of glutathione peroxidase 4 (GPX4), a glutathione peroxidase with activity dependent on GSH and selenium. This leads to an overproduction of intracellular lipid ROS, ultimately damaging the cell membrane. Solute carrier family 7 member 11 (SLC7A11), a protein closely associated with glutathione (GSH) synthesis, facilitates the biosynthesis of GSH by exchanging intracellular glutamate for extracellular cysteine. Consequently, the GPX4-SLC7A11-GSH axis is deemed a crucial regulatory node in ferroptosis [9,10]. Furthermore, nuclear factor erythroid 2 related factor 2 (Nrf2), a transcription factor responsive to oxidative stress, can augment the cell’s resistance to ferroptosis by elevating the expression of GPX4 and SLC7A11 [11–13].

In recent years, ferroptosis has emerged as a focal point in the study of liver fibrosis. Ferroptosis was observed in nonalcoholic fatty liver disease (NAFLD), which is characterized by gradual development of liver fibrosis, and ferroptosis inhibitors could efficiently prevent ferroptosis and mitigate NAFLD [14,15]. The occurrence of ferroptosis was also detected in acetaminophen-induced acute liver injury and CCl_4_-induced liver fibrosis, and blocking ferroptosis alleviated fibrosis [16,17]. Yes-associated protein inhibition ameliorated hepatitis B virus-related liver fibrosis by decreasing ferroptosis [18]. In addition, in the parasite-induced ferroptosis studies, *Toxoplasma gondii* induced ferroptosis to promote hippocampal and retinal damage in mice, and *Plasmodium* induced host liver Kuffer cell ferroptosis to promote infection [19,20]. The occurrence of ferroptosis during *C. sinensis* infection and its role in the development of liver fibrosis induced by *C. sinensis* remain ambiguous.

In the present study, we investigated the role of ferroptosis in *C. sinensis*-induced liver fibrosis. The occurrence of ferroptosis were detected in *C. sinensis*-infected C57BL/6 mouse and *C. sinensis* ESPs-stimulated AML12 cells. Furthermore, ferroptosis inhibitor was used to intervene in *C. sinensis* infected mice, and the molecular markers of ferroptosis, body weight, liver injury, parasite burden, collagen fiber deposition, pro-inflammatory cytokines, transforming growth factor (TGF)-β, and phosphorylation level of Smad2/3 were analyzed. In addition, proinflammatory cytokine production and cell viability were also examined in the ESPs-co-incubated AML12 cells treated with Fer-1.

## 2. Results

### 2.1 *C. sinensis* infection caused liver ferroptosis in mice

**Fig 1:**
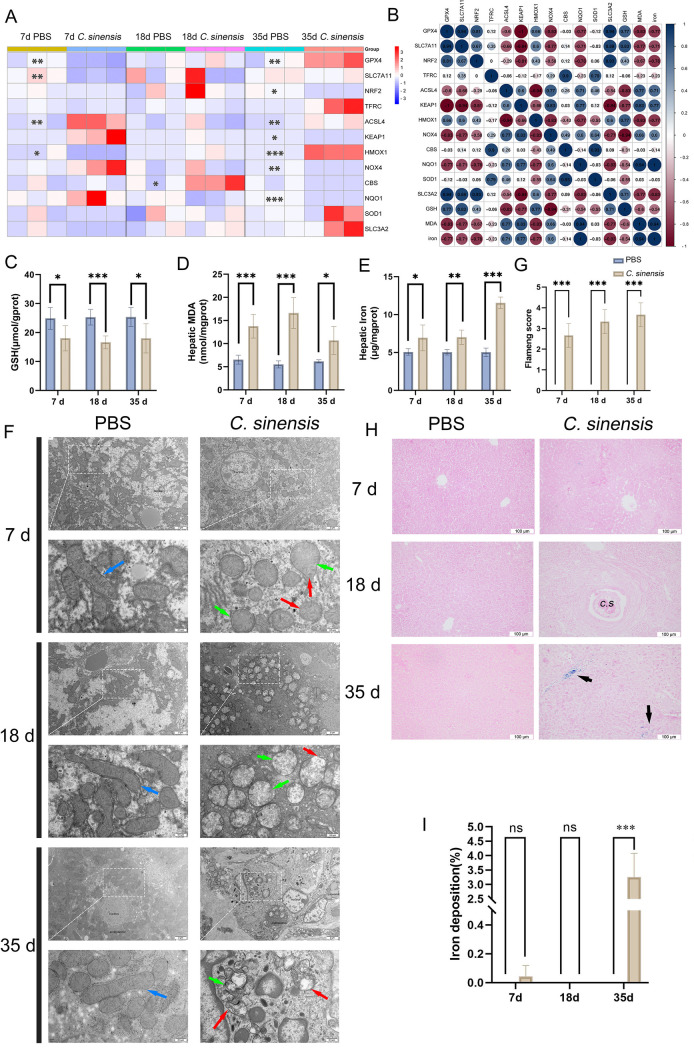
Liver ferroptosis caused by *C. sinensis* infection in mice **.** Mice were infected with *C. sinensis* metacercariae orally, and were euthanized at 7 d.p.i, 18 d.p.i, and 35 d.p.i, respectively. Liver samples were collected for the detection of ferroptosis indicators (n = 15 mice were euthanized per group at each time point). (A) The mRNA expression of ferroptosis-related genes were detected by RT-qPCR, each box represents a single individual, and close to blue represents low level of mRNA expression, close to red represents high level of mRNA expression. (B) Spearman correlation analysis of various ferroptosis indicators in mice was shown by heatmap, circle size represents correlation size, close to dark blue represents positive correlation, and close to dark red represents negative correlation. (C) GSH content of liver in mice was detected. (D) The MDA content of liver in mice was detected. (E) The total iron content of liver in mice was detected. (F) Transmission electron microscopy was used to analyze the ultrastructural changes of mice liver mitochondria, blue arrows point to mitochondria with normal morphology, green arrows point to mitochondria with reduced cristae, and red arrows to mitochondria with membrane damage (The scale bar of the original image and the local magnified image are 2μm and 0.5μm respectively.). (G) Flameng score was used to evaluate the ultrastructure of liver mitochondria in mice. (H) Prussian blue staining was used to visualize liver iron deposition, and the blue marked by black arrows is the iron deposition part (scale bars = 100μm). (I) Semi-quantitative analysis of iron deposition in (H). Data are derived from at least three biologically independent mice per group; *p < 0.05, **p < 0.01, ***p < 0.001, ns means no significant difference.

Our analysis of the gene expression related to ferroptosis, using qPCR, demonstrated that in mice infected with *C. sinensis*, there was a substantial reduction in the GPX4 and SLC7A11 genes; the decrease measured at 63.4% and 74.9% respectively, at 7 days post-infection (d.p.i) compared to the control group treated with PBS (Fig 1A). Interestingly, the expression of GPX4 at 35 d.p.i was notably increased by 168% (Fig 1A). Spearman’s correlation analysis suggested a strong correlation between the occurrence of ferroptosis and the expression of GPX4, SLC7A11, and Nrf2 at the 7 d.p.i (Fig 1B). We also measured the levels of GSH, MDA, and total iron in liver tissues. Compared to the PBS group, the levels of GSH in the livers of *C. sinensis*-infected mice were consistently decreased at 7, 18, and 35 d.p.i (Fig 1C), while there was a significant accumulation of MDA (Fig 1D) and a substantial increase in liver iron content (Fig 1E). Additionally, transmission electron microscopy examination of the ultrastructure of hepatocyte mitochondria revealed various abnormalities in the livers of *C. sinensis*-infected mice at 7, 18, and 35 d.p.i, such as a reduction in cristae, the disruption of cristae structure, increased volumes, and mitochondrial membrane damage (Fig 1F and 1G). Furthermore, iron deposition was observed in the livers of mice at 35 d.p.i with *C. sinensis* (Fig 1H and 1I). Collectively, these results suggested that *C. sinensis* infection could induce ferroptosis in the livers of mice.

### 2.2 *C. sinensis* ESPs facilitated ferroptosis in AML12 cells

**Fig 2:**
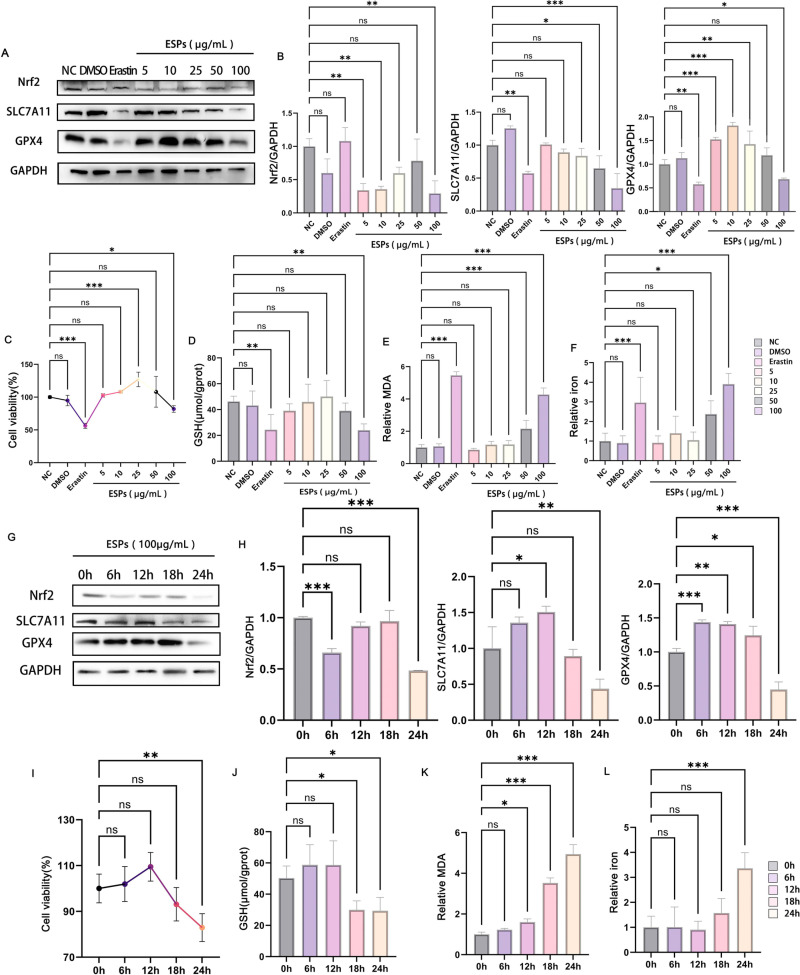
*C. sinensis* ESPs facilitated ferroptosis in AML12 cells. AML12 cells were stimulated with different concentrations of *C. sinensis* ESPs (5 μg/mL, 10 μg/mL, 25 μg/mL, 50 μg/mL, 100 μg/mL) for 24h, and Erastin (ferroptosis inducer, 10 mM, dissolved in DMSO) was set as positive control. (A) Nrf2, SLC7A11, GPX4 expression in AML12 cells were detected by western blot. (B) Relative gray values of western blot in (A) were analyzed by ImageJ software. (C) Cell viability was detected by CCK8 assay. (D) GSH content in AML12 was detected. (E) MDA content in AML12 was detected. (F) Total iron content in AML12 was measured. AML12 cells were stimulated with *C. sinensis* ESPs at a concentration of 100 μg/mL for 0h, 6h, 12h, 18h, 24h. (G) Nrf2, SLC7A11, GPX4 expression in AML12 cells were detected by western blot. (H) Relative gray values of western blot in (G) were analyzed by ImageJ software. (I) Cell viability was detected by CCK8 assay. (J) GSH content in AML12 was detected. (K) Relative MDA content in AML12 was detected. (L) Relative total iron content in AML12 was measured. Data are derived from at least three independent cell wells within one experiment; *p < 0.05, **p < 0.01, ***p < 0.001, ns means no significant difference.

AML12 cells were treated with ESPs, analogous to the ferroptosis inducer Erastin, the expression of SLC7A11 was inhibited by *C. sinensis* ESPs in a dose-dependent manner when compared with negative and DMSO control, and a significant reduction was observed in the cells stimulated with 100 μg/mL ESPs in particular (Fig 2A and 2B). The expression of Nrf2 was gradually increased in 5–50 μg/mL ESPs treatment groups, and peaked at 50 μg/mL ESPs, but decreased in 100 μg/mL ESPs treatment group (Fig 2A and 2B). The GPX4 expression was greatly increased in 5, 10 and 25 μg/mL ESPs treatment groups, but significantly reduced in 100 μg/mL ESPs treatment group, when compared with that of negative control group (Fig 2A and 2B). AML12 cells proliferated when stimulated by 5–25 μg/mL ESPs, and the viability of AML12 cells was significantly increased at 25 μg/mL. Similar with Erastin positive control group, AML12 cells viability was inhibited by 100 μg/mL ESPs (Fig 2C). Erastin significantly inhibited GSH level, and similarly GSH was significantly decreased in 100 μg/mL ESPs-stimulated cell compared with negative control group (Fig 2D). Similar to the Erastin positive control group, the MDA and iron level were significantly increased in the cells stimulated with 100 μg/mL ESPs compared to the negative control group (Fig 2E and 2F). Then cells were treated with a concentration of 100 μg/mL ESPs for different times and the protein level changes were detected by western blot. The results showed that Nrf2, SLC7A11 and GPX4 were significantly decreased in cells stimulated with ESPs at 24h (Fig 2G and 2H). In addition, CCK8 results showed that the viability of AML12 cells was significantly decreased at 24h compared with the control group (Fig 2I). Subsequently, we measured GSH, MDA, and total cellular iron at these different time points, the results showed that the levels of GSH were greatly decreased at 18h and 24h after stimulation, while the levels of MDA and total cellular iron were increased in a time-dependent manner, particularly at 24h (Fig 2J, 2K and 2L). These results suggest that ferroptosis can be induced by ESPs treatment in AML12 cells.

### 2.3 Inhibition of ferroptosis induced by *C. sinensis* ESPs alleviated cell viability

**Fig 3:**
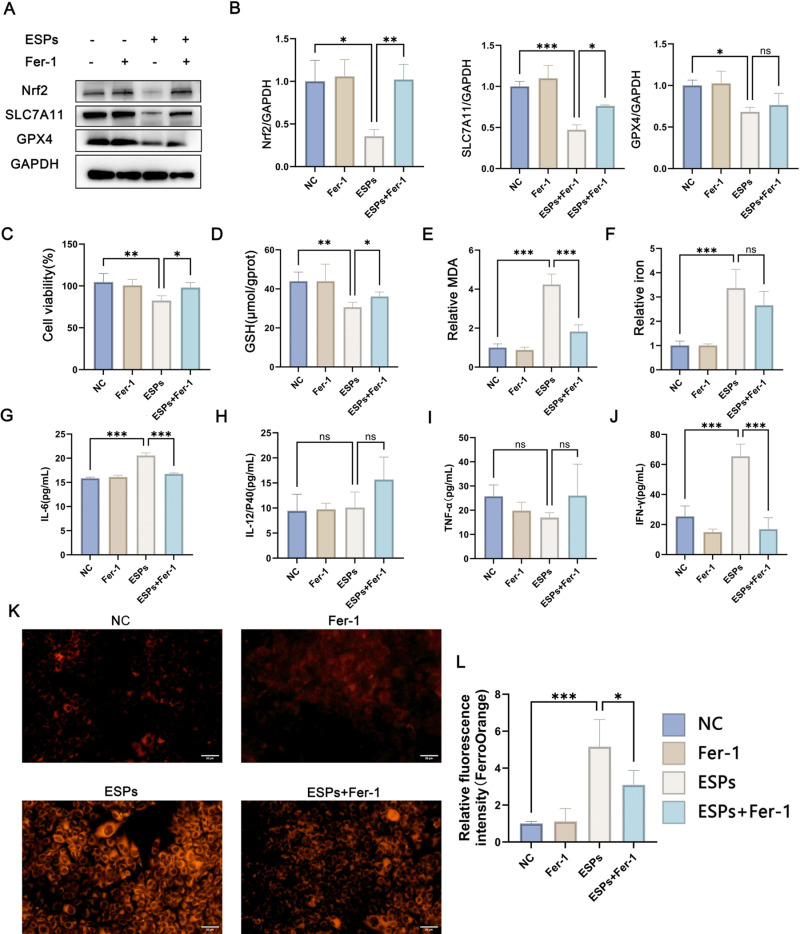
Inhibition of ferroptosis alleviated cell viability induced by *C. sinensis* ESPs. AML12 cells were pre-treated with 5 μM Fer-1 (ferroptosis inhibitor) for 1h, and then stimulated with 100 μg/mL ESPs for 24h. (A) Nrf2, SLC7A11, GPX4 expression in AML12 cells were detected by western blot. (B) Relative gray values of western blot in (A) were analyzed by ImageJ software. (C) Cell viability was detected by CCK8 assay. (D) The content of reduced GSH in AML12 cells was detected. MDA (E) and total cellular iron (F) content in AML12 cells were detected. The productions of IL-6 (G), IL-12 (H), TNF-α (I) and IFN-γ (J) in cell culture supernatant were detected by ELISA. (K) FerroOrange probe was used to detect the Fe2+ levels in AML12 cells (scale bar = 20μm). (L) Fluorescence intensity levels were quantified using ImageJ software. Data are derived from at least three independent cell wells within one experiment; *p < 0.05, **p < 0.01, ***p < 0.001, ns means no significant difference.

The expressions of SLC7A11, Nrf2 and GPX4 were greatly inhibited by ESPs, but Fer-1 pre-treatment can significantly restore the expressions of SLC7A11, Nrf2 compared to the ESPs group, while GPX4 expression were also increased, but not statistically significant (Fig 3A and 3B). In addition, the decreased cell viability caused by ESPs was significantly alleviated by Fer-1 pretreatment (Fig 3C). Compared with that of ESPs group, the cellular GSH expression was elevated after Fer-1 pretreatment, in contrast the MDA expression was decreased (Fig 3D and 3E). There was no statistical difference in total cellular iron levels induced by ESPs with or without Fe-1 pre-treatment (Fig 3F). FerroOrange staining (Orange-red fluorescence) of the ferrous probe demonstrated intracellular Fe^2+^ levels. In the negative and Fer-1 groups, low orange-red fluorescence was observed, while in ESPs group high level of orange-red fluorescence was detected, indicating elevated intracellular Fe^2+^ levels. However, the increase in Fe^2+^ content caused by ESPs stimulation was alleviated after pre-treatment with Fer-1 (Fig 3K and 3L). Additionally, cytokine detection showed significant increases in IL-6 and IFN-γ levels following ESPs treatment, whereas Fer-1 pre-treatment mitigated these elevations of both IL-6 and IFN-γ (Fig 3G–3J). Furthermore, we also detected the changes in hepatocyte TGF-β transcription levels, but neither ESPs treatment nor Fer-1 treatment could cause significant differences in hepatocyte TGF-β transcription levels (S4F Fig). These results further illustrate that *C. sinensis* ESPs promote hepatocyte injury by inducing ferroptosis.

Furthermore, supplementing with the GSH synthesis precursor N-Acetylcysteine (NAC) also mitigated the ESPs-induced ferroptosis, as GPX4 and SLC7A11 expressions were increased (S2A and S2B Fig), cell viability was improved (S2C Fig), the production of GSH was recovered (S2D Fig), and the level of MDA was greatly decreased (S2 Fig E). Moreover, pre-treatment with Tert-butyl hydroquinone (tBHQ), the Nrf2 agonist, alleviated ESPs-triggered ferroptosis, evidenced by increased expressions of Nrf2 and SLC7A11 (S3A and S3B Fig), improved cell viability (S3C Fig), recovered level of GSH (S3D Fig), decreased MDA (S3E Fig). Meanwhile, the Nrf2 nuclear import was effectively promoted by tBHQ pre-treatment (S3F-H Fig). We co-cultured hepatocytes and hepatic stellate cells (HSCs), and performed ferroptosis rescue experiments. The results showed that the inhibition of ferroptosis inhibited the activation of HSCs in the co-culture system, and Fer-1 treatment significantly reduced the expression of α-SMA (S4A-E Fig-E).

### 2.4 Fer-1 inhibited *C. sinensis-*induced liver ferroptosis

**Fig 4:**
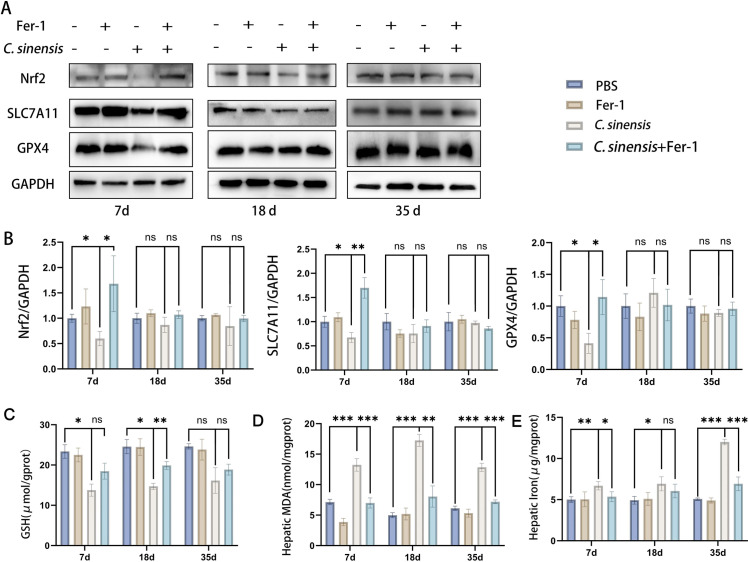
Fer-1 inhibited *C. sinensis*-induced liver ferroptosis **.** Mice infected with *C. sinensis* metacercariae were treated with Fer-1 (1 mg/kg) by intraperitoneal injection, and euthanized at 7 d.p.i, 18 d.p.i, 35 d.p.i (n = 15 mice were euthanized per group at each time point). (A) Nrf2, SLC7A11, GPX4 expressions in liver were detected by western blot. (B) Relative gray values of western blot in (A) were analyzed by ImageJ software. (C) Reduced GSH content of liver in mice was detected. (D) MDA content of liver in mice was detected. (E) Total iron content of liver in mice was detected. Data are derived from at least three biologically independent mice per group; *p < 0.05, **p < 0.01, ***p < 0.001, ns means no significant difference.

**Fig 5:**
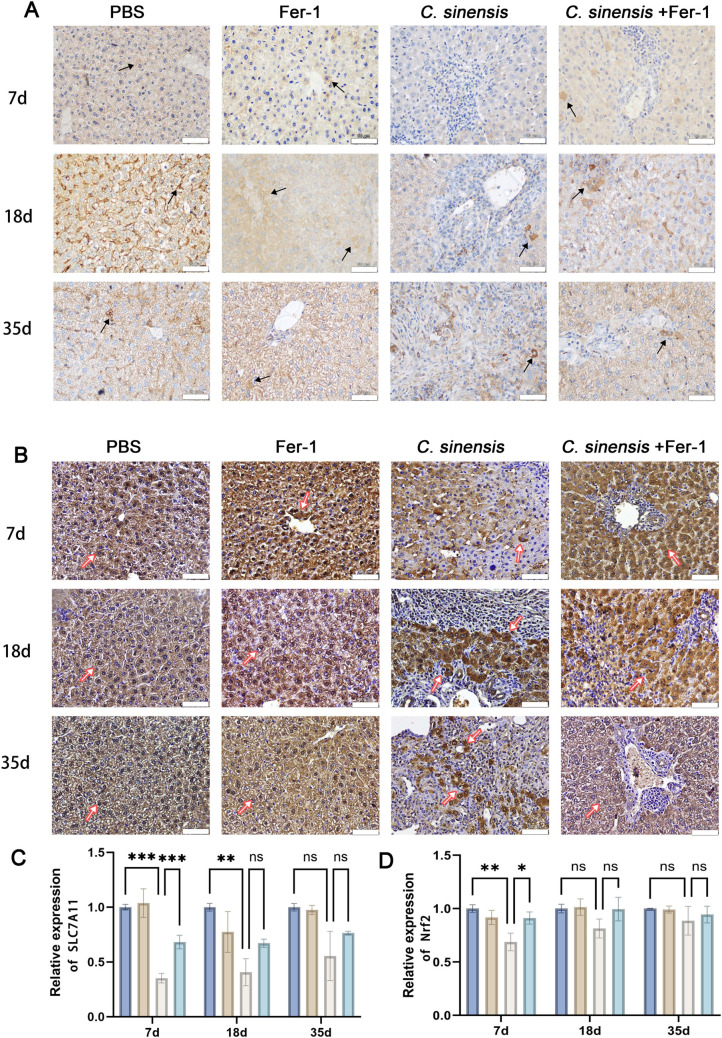
Fer-1 increased SLC7A11 and Nrf2 expression to resist *C. sinensis*-induced ferroptosis. Mice infected with *C. sinensis* metacercariae were treated with Fer-1 (1 mg/kg) by intraperitoneal injection, and euthanized at 7 d.p.i, 18 d.p.i, 35 d.p.i (n = 15 mice were euthanized per group at each time point). (A) Liver sections of mice of each group were immunohistochemically stained for SLC7A11 with brown staining (Area indicated by black arrow, scale bar = 50μm). (B) Liver sections of mice in each group were immunohistochemically stained for Nrf2 with brown staining (Area indicated by red arrow, scale bar = 50μm). (C) Semi-quantitative analysis of the sections by ImageJ software to determine the level of SLC7A11 expression. (D) Semi-quantitative analysis of the sections by ImageJ software to determine the level of Nrf2 expression. Data are derived from at least three biologically independent mice per group; *p < 0.05, **p < 0.01, ***p < 0.001, ns means no significant difference.

The Nrf2, SLC7A11 and GPX4 expressions were significantly decreased in *C. sinensis* group at early stage (7 d.p.i) compared to PBS group, indicating ferroptosis occurrence, while there was almost no change at middle and late stages (18 d.p.i, 35 d.p.i). However, Fer-1 pre-treatment can reduce *C. sinensis*-induced ferroptosis, increased expressions of Nrf2, SLC7A11 and GPX4 were detected when compared to *C. sinensis* infection group (Fig 4A and 4B). Then GSH, MDA and total iron in livers were detected. Liver GSH levels in *C. sinensis*-infected mice were significantly lower than that in PBS group at 7 d.p.i and 18 d.p.i, but Fer-1 treatment elevated the liver GSH levels (Fig 4C). In addition, the liver MDA levels were significantly reduced in *C. sinensis*+Fer-1 group compared to *C. sinensis* group at all time points (Fig 4D). The levels of liver iron were increased by *C. sinensis*, but significantly decreased at 7 d.p.i and 35 d.p.i in the *C. sinensis*+Fer-1 group (Fig 4E). Immunohistochemistry staining was used to detect the expression of SLC7A11 and Nrf2 in mice liver. Consistent with the results of Fig 4A and 4B, the expression of SLC7A11 was significantly decreased in *C. sinensis* group compared to PBS group, treatment of Fer-1 rescued the decreased expression of SLC7A11 (Fig 5A and 5C). In comparison to the PBS group, the area of positive Nrf2 expression was significantly decreased in the *C. sinensis* group, while treatment with Fer-1 rescued the early reduction of Nrf2 expression (Fig 5B and 5D). In addition, we examined the production of hepatic Fe^2+^ and the expressions of related proteins heme oxygenase-1 (HO-1) and Transferrin receptor protein 1 (TFRC) in mice. *C. sinensis* infection significantly increased Fe^2+^ content and HO-1 and TFRC expression at 35 d.p.i compared with PBS group (S5 Fig). These increases were significantly alleviated by treatment with Fer-1 (S5 Fig). These results demonstrated that Fer-1 inhibited liver ferroptosis induced by *C. sinensis.*

### 2.5 Inhibition of ferroptosis mitigated liver injury caused by *C. sinensis* infection

**Fig 6:**
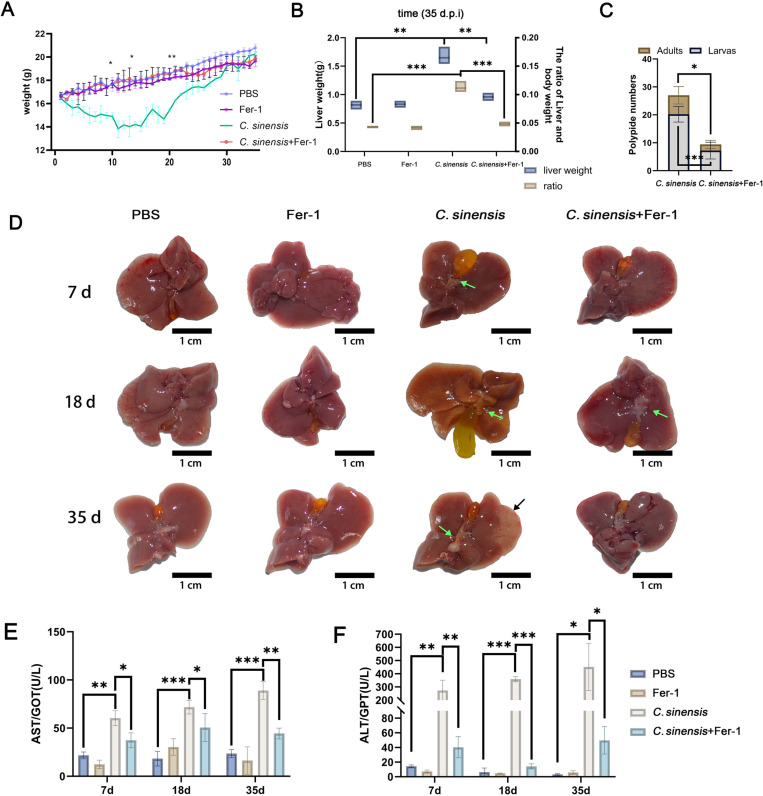
Inhibition of ferroptosis mitigated liver injury caused by *C. sinensis* infection. Mice infected with *C. sinensis* metacercariae were treated with Fer-1 (1mg/kg) by intraperitoneal injection, and euthanized at 7 d.p.i, 18 d.p.i, 35 d.p.i (n = 15 mice were euthanized per group at each time point). (A) Body weight changes of mice (PBS, Fer-1, *C. sinensis*, *C. sinensis* + Fer-1) from 1 to 35 day were plotted as a line graph. (B) Liver weight and ratio of liver weight to body weight at 35 d.p.i in each group were recorded, blue bars = liver weight, yellow bars = liver/body ratio. (C) The number of intrahepatic parasites of each mouse was counted in *C. sinensis* and *C. sinensis* + Fer-1 groups. (D) Liver lesion of mice in PBS, Fer-1, *C. sinensis* and *C. sinensis*+Fer-1 groups were observed, green arrows refer to the cholangiectasis that occurred after *C. sinensis* infection, and black arrow indicated liver fibrosis (scale bar = 1 cm). (E F) The levels of serum AST and ALT levels in mice were detected. Data are derived from at least three biologically independent mice per group; *p < 0.05, **p < 0.01, ***p < 0.001, ns means no significant difference.

**Fig 7:**
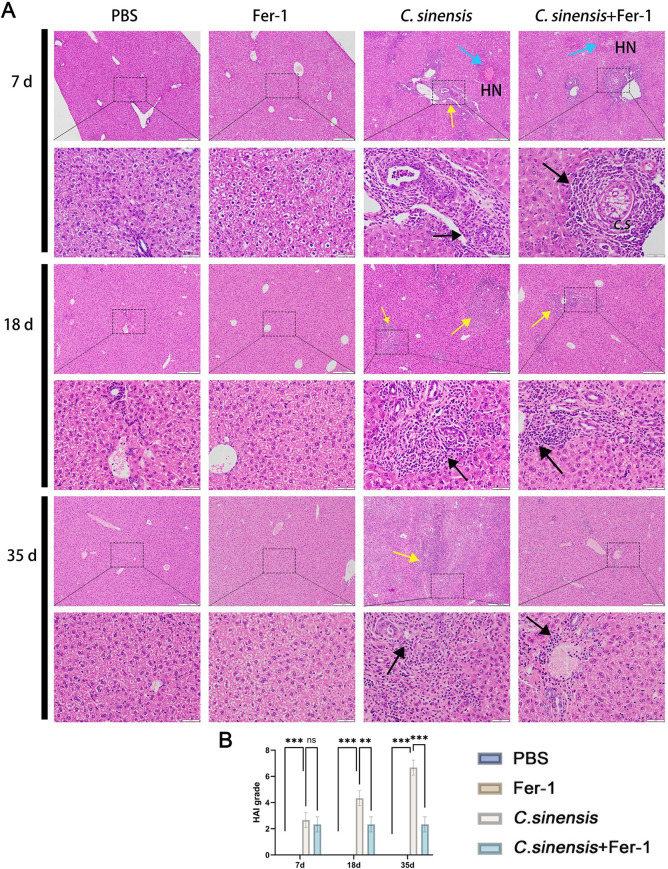
Fer-1 alleviated liver lesions caused by *C. sinensis.* (A) Liver tissue sections were prepared and stained with H&E. The black arrows indicated inflammatory cell infiltration, the yellow arrows indicated the connective tissue hyperplasia area, the blue arrows indicated the necrotic area (HN: hepatic cells necrotic, C.*S*: *C. sinensis*) (scale bar = 100μm; scale bar = 20μm). (B) The degree of liver injure was calculated by hepatic histological activity index (HAI). Data are derived from at least three biologically independent mice per group; **p < 0.01, ***p < 0.001, ns means no significant difference.

The body weight of mice in *C. sinensis* group exhibited a significant reduction compared to the PBS group, but Fer-1 treatment significantly alleviated the weight loss caused by *C. sinensis* infection (Fig 6A). Compared with the PBS group, the liver weight of mice in *C. sinensis* group was increased significantly, but this increase was significantly alleviated by Fer-1 treatment (18.4% ~ 29.2%) (Fig 6B). Furthermore, treatment with Fer-1 significantly reduced the number of parasites in liver (average of 13 larvae and 4.6 adults per mouse) when compared to that of *C. sinensis* group (Fig 6C). Furthermore, we conducted *in vitro* toxicity tests of Fer-1 on adult *C. sinensis*. The results revealed that Fer-1 exhibits no discernible toxic effects on this species (S6 Fig). Liver lesions were observed in *C. sinensis*-infected mice and exacerbated in a time-dependent manner, including bile duct hyperplasia and thickening, jaundice, and connective tissue hyperplasia. The mice in *C. sinensis*+Fer-1 group exhibited mild bile duct thickening without pronounced jaundice or connective tissue hyperplasia (Fig 6D). Histological examination showed abundant inflammatory cell infiltration, hepatocellular necrosis foci, pseudolobular formation in the livers of *C. sinensis*-infected mice at 18 d.p.i; and extensive connective tissue hyperplasia in livers of mice at 35 d.p.i in *C. sinensis* group (Fig 7A). Compared to the *C. sinensis* group, the liver lesions were diminished, inflammation was reduced, and connective tissue was decreased in the mice of *C. sinensis*+Fer-1 group (Fig 7A). The hepatic histological activity index (HAI) score of liver tissue sections revealed that the liver injury in the *C. sinensis*+Fer-1 group was significantly alleviated compared to that in the *C. sinensis* group (Fig 7B). Finally, we detected the liver damage indicators AST and ALT, infection with *C. sinensis* caused increases of AST and ALT in mice serum, while treatment with Fer-1 significantly decreased the AST content in mice serum at 35 d.p.i (50.1%) (Fig 6E), Fer-1 treatment also significantly decreased the ALT contents in mice serum at 7 d.p.i, 18 d.p.i, 35 d.p.i (85.3%, 96.0%, 88.9%) (Fig 6F). The above results demonstrated that *C. sinensis*-caused ferroptosis contributed to the hepatic injury, and ferroptosis inhibitor Fer-1 can relieve this injury.

### 2.6 Inhibition of ferroptosis alleviated liver fibrosis caused by *C. sinensis* infection

**Fig 8:**
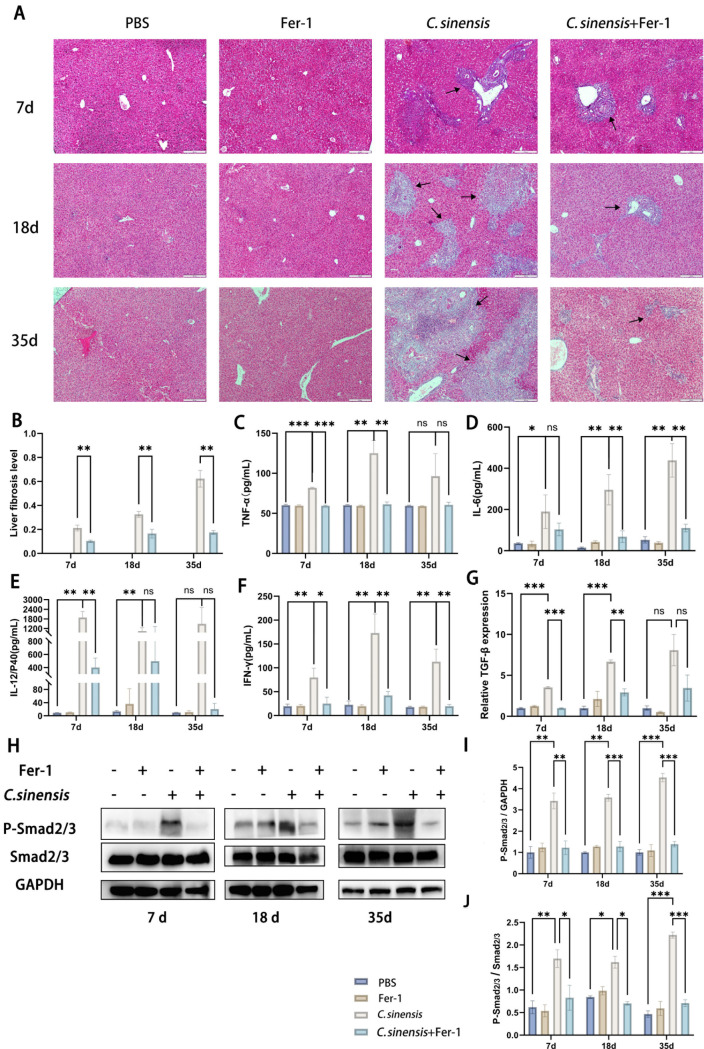
Inhibition of ferroptosis alleviated liver fibrosis caused by *C. sinensis.* Mice infected with *C. sinensis* metacercariae were treated with Fer-1 (1mg/kg) by intraperitoneal injection, and euthanized at 7 d.p.i, 18 d.p.i, 35 d.p.i (n = 15 mice were euthanized per group at each time point). (A) The deposition of collagen fibers in the liver was determined by Masson staining, and the deposition of collagen fibers was visualized by blue, with black arrows indicating the area of collagen fiber deposition (scale bar = 100μm). (B) Collagen depositions from each specimen were estimated using ImageJ software. (C–F) TNF-α, IL-6, IL-12 and IFN-γ in mice serum were detected by ELISA. (G) RT-qPCR was used to detect mRNA expression of TGF-β in total RNA from mice liver. (H-J) The phosphorylation of Smad2/3 in liver were analyzed by western blot. Data are derived from at least three biologically independent mice per group; *p < 0.05, **p < 0.01, ***p < 0.001, ns means no significant difference.

Liver fibrosis levels of mice in each group were evaluated by Masson staining of liver collagen fibers. After euthanasia at 7 d.p.i, 18 d.p.i, and 35 d.p.i, the liver was fixed with neutral formalin, prepared into paraffin sections, and stained using a Masson staining kit. Using microscopy to observe sections, blue-stained collagen fiber deposits were observed in the liver of *C. sinensis* group mice. Compared with the *C. sinensis* group, the fibrotic area in the *C. sinensis *+ Fer-1 group was significantly reduced at all time points (Fig 8A and 8B). The TNF-α, IL-6, IL-12 and IFN-γ of *C. sinensis* group were significantly increased, but greatly decreased in *C. sinensis*+Fer-1 group (Fig 8C-8F). Compared with *C. sinensis* group, the upregulation of hepatic TGF-β mRNA expression was inhibited in *C. sinensis*+Fer-1 group and there were statistically significant differences at 7 d.p.i and 18 d.p.i (Fig 8G). In addition, the Smad2/3 phosphorylation level was significantly decreased in *C. sinensis*+Fer-1 group compared with that of *C. sinensis* group mice (Fig 8H-8J).

**Fig 9:**
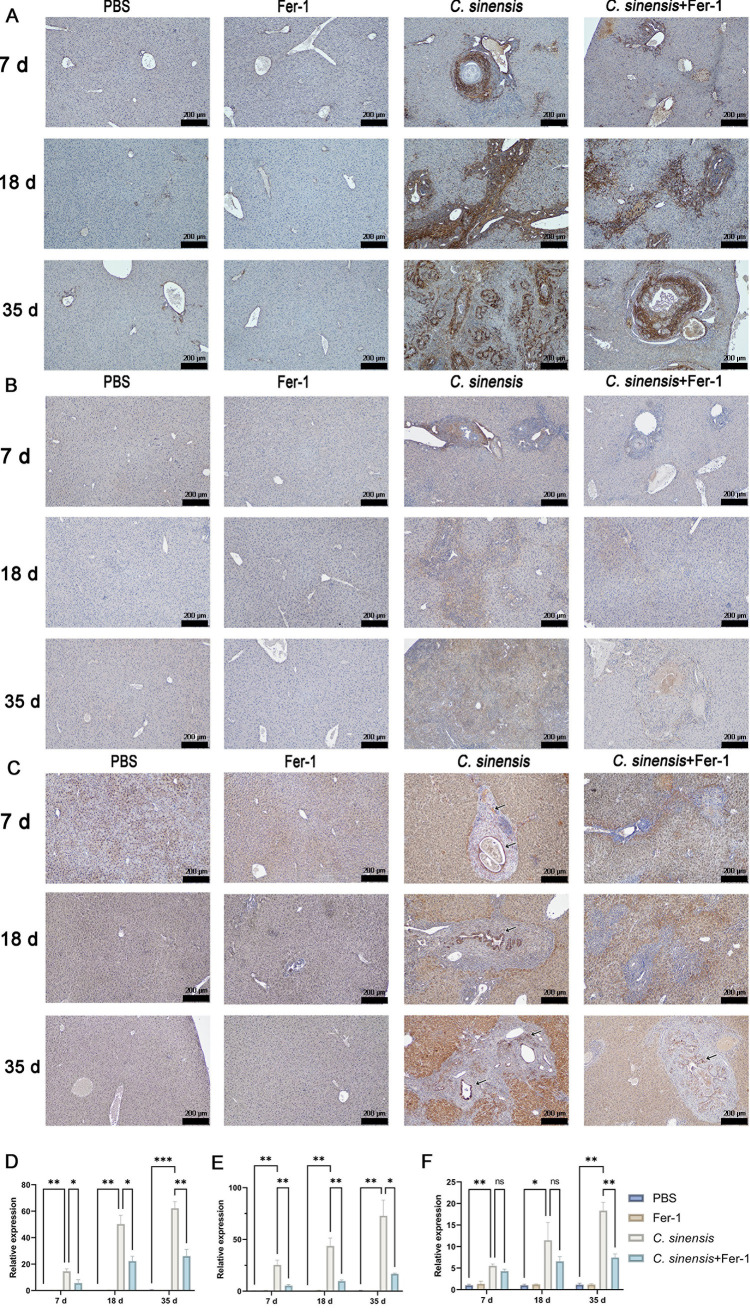
Inhibition of ferroptosis alleviated the expression of hepatic **α-SMA, collagenⅠ, and collagen Ⅲ induced by *C. sinensis* in mice.** (A) Liver sections of mice of each group were immunohistochemically stained for α-SMA with brown staining (scale bar = 200μm). (B) Liver sections of mice of each group were immunohistochemically stained for collagen Ⅰ with brown staining (scale bar = 200μm). (C) Liver sections of mice of each group were immunohistochemically stained for collagen Ⅲ with brown staining (scale bar = 200μm). (D-F) Semi-quantitative analysis of the sections by ImageJ software to determine the level of α-SMA, collagen Ⅰ, and collagen Ⅲ expression. Data are derived from at least three biologically independent mice per group; *p < 0.05, **p < 0.01, ***p < 0.001, ns means no significant difference.

Immunohistochemical staining for fibrosis markers α-SMA, collagen I, and collagen III. After infection with *C. sinensis*, the expression of α-SMA, collagen I, and collagen III in the liver of mice significantly increased (7 d.p.i, 18 d.p.i, and 35 d.p.i). After treatment with Fer-1, the expression of α-SMA and collagen I significantly decreased (7 d.p.i, 18 d.p.i, and 35 d.p.i), and the expression of collagen III at 35 d.p.i was significantly lower than that in the *C. sinensis*-infected group of mice. (Fig 9A-G). These results indicate *C. sinensis*-caused TGF-β/Smad pathway activation and liver fibrosis were inhibited the and alleviated by the Fer-1 treatment.

## 3. Discussion

Programmed cell death triggers inflammation via membrane permeability changes, recruiting immune cells and activating HSCs to drive fibrosis [6,21]. Iron-rich HSCs and pathological iron overload implicate ferroptosis in this process [22–25]. Previous study has demonstrated that *C. sinensis* can activate HSCs to promote liver fibrosis by secreting virulence factors such as Lysophospholipase A and phospholipase A2 [26,27]. In addition, *C. sinensis* infection causes accumulations of lipid peroxidation products such as Hydroxynonenal and MDA, accompanied by hepatic iron deposition, suggesting a potential role of ferroptosis in *C. sinensis*-induced liver fibrosis [28,29]. It was speculated that ferroptosis might be involved in the process of liver fibrosis induced by *C. sinensis*.

Ferroptosis, a form of programmed cell death dependent on ROS, is characterized by iron overload, lipid peroxidation and absence of mitochondrial cristae [30]. Excess free iron in cells accelerates lipid ROS generation by driving Fenton reaction., and excess lipid ROS subsequently reacts with polyunsaturated fatty acids to generate lipid hydroperoxides, which are ultimately oxidized to MDA, a common end product of lipid peroxidation [31]. Mitochondria are the center of energy metabolism in cells and one of the main sources of ROS in cells. Blocking abnormal ROS production in mitochondria is considered to prevent ferroptosis. Abnormalities in function are reflected in changes of morphological structure, so cells undergoing ferroptosis exhibit abnormal mitochondria, including swelling or shrinkage, reduced cristae, cristae rupture, and membrane rupture. Distinct from apoptosis, cells endured ferroptosis release Damage-associated molecular patterns (DAMPs), which can incite tissue damage through inflammatory responses [32,33]. Thus, ferroptosis is thought to be associated with multiple disease processes, including cardiovascular, metabolic, neurological, and hepatic conditions [34–36]. In pathogen-induced diseases, ferroptosis is also regarded as an important process. For instance, the hepatitis C virus in viruses, the *Mycobacterium tuberculosis* in bacteria, and the *Toxoplasma gondii* and *Plasmodium* in protozoa can all induce host cell ferroptosis which is characterized by MDA accumulation and mitochondrial structure damage, thereby promoting immune escape or aggravating tissue injury [20,37–39]. We found that *C. sinensis* infection in mice caused hepatic ferroptosis, evidenced by iron overload, lipid peroxidation, and mitochondrial damage. In AML12 cells, high-concentration ESPs from *C. sinensis* reduced viability, increased iron and MDA, while low concentrations promoted proliferation—possibly due to ESPs’ complex composition and cellular tolerance. This makes the role of ESPs on Erastin’s action somewhat different. Thus, *C. sinensis* likely induces hepatocyte ferroptosis via ESPs, linking ferroptosis to parasite-driven liver fibrosis.

Ferroptosis can be mainly activated by two pathways, one is the transporter-dependent pathway, such as via inhibiting system xc-, and another is the enzyme-regulated pathway, such as by directly inhibiting GPX4, inducing lipid peroxidation, or inducing mitochondrial dysfunction [40]. As a component of system xc-, the cystine/glutamate antiporter, SLC7A11 is critical for cystine transport and subsequent GSH synthesis, reducing SLC7A11 activity will directly lead to impaired transport of cystine into cells, and thus affect GSH synthesis [41]. Erastin, for example, induces ferroptosis by targeting inhibition of SLC7A11 activity to block GSH synthesis and induce intracellular lipid ROS accumulation [42]. Impaired GSH synthesis results in the inactivation of GPX4, a critical enzyme that quenches lipid peroxidation. When unchecked, this process culminates in the production of phospholipid hydroperoxides and cell death [43]. We observed that the GSH content in the liver of *C. sinensis*-infected mice was decreased, which corresponded to a decrease in GPX4 activity at 7 d.p.i. And high concentrations of ESPs promoted similar results. NAC, a well-known precursor of GSH, rescued AML12 cells from ferroptosis induced by ESPs. Previous studies have showed that ESPs promoted collagen I secretion in HuCCT cells by promoting ROS generation, and treatment with NAC reduced the expression of collagen I [5]. This suggests that *C. sinensis* infection is likely to promote severe damage by ferroptosis through the consumption of GSH. In addition, downregulation of SLC7A11 expression caused by *C. sinensis* infection in mouse liver and ESPs stimulation in AML12 cells further corroborated the reduction in cellular GSH synthesis. Nrf2, a pivotal regulator in the response to oxidative stress, translocates to the nucleus under conditions of redox imbalance to modulate antioxidant genes, such as SLC7A11, when Nrf2 cannot be restored to its normal state beyond the threshold of lipid peroxidation cell recovery, it loses its regulation of downstream antioxidant gene expression [44]. We observed that Nrf2 expression was decreased in the liver of *C. sinensis*-infected mice and ESPs-stimulated AML12 cells, and a decrease in the nuclear entry rate of Nrf2 was observed in AML12 cells. Treatment of AML12 cells with tBHQ, a Nrf2 agonist, enhanced resistance to ESP-induced ferroptosis, by promoting Nrf2 nuclear entry and increasing SLC7A11 and GPX4 expression. Our findings indicate that *C. sinensis* infection can induce liver ferroptosis, which is associated with diminished Nrf2, GPX4, and SLC7A11 expression.

Ferroptosis is known to provoke inflammatory responses, with research showing that inhibiting ferroptosis can reduce the release of IL-6 and COX-2 in ulcerative colitis [45,46]. Pro-inflammatory cytokines, such as IL-1β and IL-6, are implicated in the progression of ferroptosis [47,48]. This inflammatory milieu can activate TGF-β, leading to the activation of hepatic stellate cells and the Smad signaling pathway, which is central to liver fibrosis [49,50]. *C. sinensis* has been shown to promote pro-inflammatory cytokine release, activating the TGF-β/Smad pathway and contributing to liver fibrosis [49,50]. This study also showed that Fer-1 pretreatment significantly reduced ESP-induced expression of IL-6 which is a potent profibrotic cytokine in AML12 cells, suggesting that targeting ferroptosis could be a viable strategy for treating *C. sinensis*-induced fibrosis. Separate, we noticed that ESPs had very little effect on TGF-β expression of AML12, which is probably a hepatocyte itself characteristic since liver TGF-β mainly originates from macrophages and HSCs [51]. To evaluate ESP-induced hepatocyte ferroptosis effects on HSCs, we used a hepatocyte-HSC coculture model [52]. Ferroptosis inhibition reduced ESP-induced α-SMA expression, suggesting hepatocyte ferroptosis contributes to HSC activation. While this simplified model provides initial insights, more complex systems incorporating immune components may better replicate the *in vivo* fibrotic microenvironment.

Targeting ferroptosis has been demonstrated to have therapeutic potential in conditions such as non-alcoholic hepatitis, with agents like Fer-1 and tBHQ showing promise in alleviating liver injury [53]. In the context of parasitic diseases, iron chelators like Deferiprone have shown efficacy in reducing inflammation and disease severity [19,20]. Deferiprone not only reduces the iron overload but also ameliorates *T. gondii*-induced retinochoroiditis by reducing retinal inflammation [39]. Fer-1 has been shown to inhibit ferroptosis and alleviate injury in models of acute lung injury and kidney disease. In lipopolysaccharide-induced acute lung injury, Fer-1 alleviated lung injury by inhibiting ferroptosis. In oxalate-induced kidney injury, treatment with Fer-1 significantly alleviated renal tubular epithelial injury, fibrosis and formation of calcium oxalate stones [54,55]. Here, our study demonstrated that Fer-1 pretreatment enhanced resistance to ESP-induced ferroptosis in AML12 cells, evidenced by reduced GSH depletion and decreased MDA and iron accumulation. *In vivo* Fer-1 treatment not only alleviated liver lesions but also reduced the parasite load in the liver of mice. Although there is no evidence that Fer-1 acted directly to clear worms, it is possible that this treatment enhanced the host resistance against the parasites and thus prevented parasite infection. This treatment also diminished the release of pro-inflammatory cytokines and the activation of the TGF-β/Smad pathway, leading to a reduction in liver fibrosis.

These results highlight the significance of ferroptosis in *C. sinensis*-induced liver inflammation and fibrosis, suggesting that developing therapeutics targeting ferroptosis could offer a novel approach to treat clonorchiasis. However, it is undeniable that this study still has limitations: Firstly, the *in vitro* model cannot fully distinguish ESP’s direct effects on HSCs from ferroptosis-mediated activation, relying on comparative analysis; Secondly, the *in vitro* models poorly replicate inflammatory/immune aspects of *C. sinensis*-induced fibrosis, an organoid models can be constructed to further investigate the molecular mechanism. Finally, given ESPs’ ferroptosis-regulating properties identified here, we recommend single-cell multi-omics studies to establish temporal models of fibrosis progression, enabling systematic analysis of spatiotemporal ferroptosis signaling.

**Fig 10:**
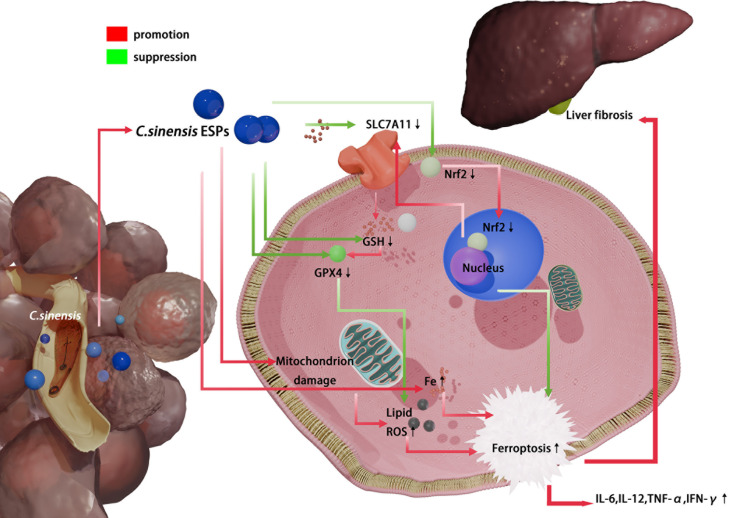
*C. sinensis* inhibits the Nrf2, SLC7A11, and GPX4 activity of hepatocyte through the secretion of ESPs, promoting GSH depletion, iron overload, leading to lipid ROS accumulation, and facilitating ferroptosis. This results in elevated levels of pro-inflammatory cytokines IL-6, IL-12, IFN-γ, and TNF-α in mice. It also promotes the activation of the liver fibrosis signaling pathway TGF-β/Smad and further enhances liver fibrosis.

In summary, *C. sinensis* infection caused iron overload and lipid peroxidation in the liver of mice, and mitochondrial morphological changes characterized by reduced cristae, showed features of ferroptosis. This can be attributed to inhibiting hepatocyte GSH synthesis, suppressing Nrf2-SLC7A11-GPX4 activity, promoting MDA accumulation and iron overload by *C. sinensis* ESPs (Fig 10). The ferroptosis inhibitor Fer-1 blocked the cytotoxicity of ESPs on AML12 *in vitro* and inhibited *C. sinensis*-induced liver ferroptosis *in vivo*, and Fer-1 also inhibited the expression of pro-inflammatory cytokines and liver fibrosis caused by *C. sinensis* infection. Our findings provide a new insight of ferroptosis in clonorchiasis treatment

## 4. Methods

### 4.1 Ethics statement

All animal experimental procedures were reviewed and approved by the Animal Welfare and Research Ethics Committee of Jilin University (IACUC permit number: 20160612). This study strictly adhered to the Guidelines for Ethical Review of Animal Welfare (National Standard GB/T 35892–2018, China).

### 4.2 Animals

Specific Pathogen Free (SPF) female C57BL/6 mice (15 ~ 16g) were purchased from Beijing HFK Bioscience CO.LTD (China).

### 4.3 Collection of *C. sinensis* metacercariae and ESPs

*C. sinensis* metacerariae were isolated from freshwater fishes which were sourced from the endemic waters of *C. sinensis* with previously described procedures [56]. ESPs were isolated from *C. sinensis* adults. Briefly, the adults were incubated at 37 °C for a duration of 6h in serum-free DMEM/F12 medium (Gibico, C11330500BT, USA) at a density of 5 parasites per mL, then the supernatant was collected [57]. The supernatant was processed through continuous centrifugations at 280 × *g* for 10 min and 2000 × *g* for 10 min, followed by filtration with a 0.22 μm filter (Sigma, SLGP033, USA).

### 4.4 Cell culture and *C. sinensis* ESPs treatment

The mouse normal hepatocyte AML12 cells were purchased from ATCC (CRL-2254, USA). These cells were cultured in DMEM/F12 medium, supplemented with 10% fetal bovine serum (FBS, Bio-Channel, BC-SE-FBS01, China) and Penicillin-Streptomycin Solution (100 units/mL penicillin and streptomycin). AML12 was maintained in a 5% CO_2_ incubator at a temperature of 37 °C. Cells were seeded at a density of 3 × 105 per well on a 6-well cell culture plate, and cultured overnight in medium containing 1% FBS, then in serum-free medium for 3h before stimulation. Subsequently, these serum-starved cells were stimulated with gradient concentrations of ESPs (5 μg/mL, 10 μg/mL, 25 μg/mL, 50 μg/mL, 100 μg/mL) for 24h and the stimulation time gradient (0h, 6h, 12h, 18h, 24h) was set to determine the optimal stimulation dose and stimulation time. In rescue experiments, cells were treated with Fer-1 (5 μM) (MCE, HY-100579, USA) [53] for 1h before ESPs treatment, and cells were collected after ESPs treatment for 24h. Erastin, a ferroptosis inducer (10 mM, dissolved in DMSO, MCE, HY-15763, USA) was set as the positive control. Cell viability was measured by using the CCK-8 assay (Selleck, B34302, USA).

### 4.5 Animal experiment

The mice were evenly and randomly allocated into two distinct groups: the PBS group and the *C. sinensis* group, with 15 mice per group at each time point. The mice in *C. sinensis* group were infected with 100 metacercariae by gavage in 200 μL phosphate buffer solution (PBS), and mice in PBS group were given 200 μL PBS. Mice were euthanized at 7 days, 18 days, and 35 days post infection.

In the treatment experiment, mice received intraperitoneal injections of Fer-1 (1 mg/kg body weight, everyday [53]) after infection with *C. sinensis* metacercariae. Mice were categorized into four groups as illustrated in S1 Fig: the PBS group, the Fer-1 treatment group, the *C. sinensis* group, and the *C. sinensis*+ Fer-1 group. These mice were euthanized at intervals of 7 days, 18 days, and 35 days post infection. Blood was collected and the serum was obtained. Livers were collected, with part of each liver fixed in neutral formalin, and the rest stored at −80 °C using for further experiments.

### 4.6 Real-Time quantitative PCR analysis

Total RNA was extracted from the liver of the aforementioned treatment group mice utilizing TRIzol reagent (Transgen, ET101, China), which was reversely transcribed into cDNA. Subsequently, the expression level of mRNA was detected by Real-Time quantitative PCR. The genes and primers employed are detailed in Table 1. Real-Time quantitative PCR was performed using BlasTaq™2 × qPCR MasterMix (abm, G891, Canada), with a program of: 95 °C for 3 min, followed by 40 cycles of 95 °C for 15 s and 60 °C for 60 s. Data were normalized to GAPDH, and all experimental mRNA levels were expressed as (delta) (delta) C/t equation [58]. And bioinformatics.com.cn was used to draw the expression heat map [59].

**Table 1:** Primer sequences used for Real-Time quantitative PCR.

Gene	Sequences (5′ to 3′)
GPX4-F	TGTGCATCCCGCGATGATT
GPX4-R	CCCTGTACTTATCCAGGCAGA
ACSL4-F	TCCTCCAAGTAGACCAACCCC
ACSL4-R	AGTCCAGGGATACGTTCACAC
CBS-F	GGAAAATTGGGAACACCCCTAT
CBS-R	CCACCCGCATTGAAGAACTCA
GAPDH-F	AGGTCGGTGTGAACGGATTTG
GAPDH-R	GGGGTCGTTGATGGCAACA
HMOX1-F	GATAGAGCGCAACAAGCAGAA
HMOX1-R	CAGTGAGGCCCATACCAGAAG
KEAP1-F	TCGAAGGCATCCACCCTAAG
KEAP1-R	CTCGAACCACGCTGTCAATCT
NOX4-F	CCTTTTACCTATGTGCCGGAC
NOX4-R	CATGTGATGTGTAGAGTCTTGCT
NQO-1-F	AGGATGGGAGGTACTCGAATC
NQO-1-R	AGGCGTCCTTCCTTATATGCTA
Nrf2-F	TCAGCGACGGAAAGAGTATGA
Nrf2-R	CCACTGGTTTCTGACTGGATGT
SLC3A2-F	TGATGAATGCACCCTTGTACTTG
SLC3A2-R	GCTCCCCAGTGAAAGTGGA
SLC7A11-F	GGCACCGTCATCGGATCAG
SLC7A11-R	CTCCACAGGCAGACCAGAAAA
SOD1-F	AACCAGTTGTGTGTGTGTCAGGAC
SOD1-R	CCACCATGTTTCTTAGAGTGAGG
TFRC-F	GTTTCTGCCAGCCCCTTATTAT
TFRC-R	GCAAGGAAAGGATATGCAGCA
TGF-β1-F	GTGTGGAGCAACATGTGGAACTCTA
TGF-β1-R	TTGGTTCAGCCACTGCCGTA

### 4.7 Transmission electron microscopy assays

The liver samples (1 mm^3^) were fixed with 2.5% glutaraldehyde at 4°C for 24h. washed three times with PBS. The liver samples were subsequently fixed using 1% osmium tetroxide for a duration of 2h. Subsequently, the liver samples underwent dehydration in a progressive ethanol and acetone series, followed by embedding in Spurr resin. Ultra-thin sections were then prepared and stained with lead citrate and uranyl acetate. A JEM1400PLUS (JEOL Company, Japan) was used to observe the tissues and capture images.

### 4.8 Western blot

Liver tissue and hepatocyte AML12 were lysed by pre-cooled RIPA lysis buffer (Yalzyme, PC101, China) containing protease inhibitor (mei5bio, MF182, 1:100, China) and phosphatase inhibitor (mei5bio, MF183, 1:100, China). The protein concentrations in the samples were measured using the BCA protein assay (Sangon, C503021, China). The western blotting procedure was conducted as previously outlined [60]. Following SDS-PAGE electrophoresis, the protein samples were electrotransferred onto polyvinylidene difluoride (PVDF) membranes via the wet transfer method. After blocking with a quick blocking solution (Yalzyme, PS108P, China) for 15 minutes, the PVDF membranes were incubated with primary antibodies overnight at 4 °C. The usage of primary antibodies was shown in S1 Table. The PVDF membranes were then washed three times with phosphate buffered solution with tween20 (PBST) and then incubated with secondary antibody (HRP-Goat Anti Rabbit IgG Antibody or HRP-Goat Anti Mouse IgG Antibody, proteintech, SA00001–1, SA00001–2, China) for 1h at room temperature. A chemiluminescence developer (Clinx scientific instrument Co. LTD, China) was used for visualization. ImageJ (Maryland Institute of Health Sciences, USA) was utilized to quantify the level of protein expression.

### 4.9 Determination of biochemical indices and cytokines

Tissue samples and cell samples were detected by the following assay kits according to the manufacturer’s instructions, respectively. Reduced Glutathione Assay Kit (Nanjing Jiancheng Bioengineering Institute, A006-2–1, China) was used to measure GSH level. The MDA Assay Kit (Nanjing Jiancheng Bioengineering Institute, A003-4–1, China) was used to measure MDA level. The level of iron was detected by the Total Iron Assay Kit (Solarbio, BC4355, China), and the ferrous ion content was measured by the Ferrous Ion Assay Kit (Solarbio, BC5415, China). Serum was collected from mice, and AST/ALT expression was detected according to the instructions of Aspartate aminotransferase assay kit (Nanjing Jiancheng Bioengineering Institute, C010-2–1, China) and Alanine aminotransferase assay kit (Nanjing Jiancheng Bioengineering Institute, C009-2–1, China), and readings were taken at a wavelength of 510 nm. The production levels of cytokines in the serum of the mice were detected using ELISA kits (IL-12/P40, EMIL12BX10; IL-6, KMC0061; TNF-α, BMS607-3; IFN-γ, BMS606-2 Thermo Fisher Scientific, USA).

### 4.10 Histopathological and immunohistochemical analysis

Liver tissue from the same anatomical lobe of each mouse was fixed in 10% neutral buffered formalin and paraffin-embedded. Serial 5 μm sections were deparaffinized (Servicebio, G1128, China), stained with H&E for histological assessment, or processed for immunohistochemistry using anti-SLC7A11 (1:250; ab307601, Abcam, USA), anti-Nrf2 (1:200; A0674, Abclonal, China) antibodies, anti-α-SMA (1:200; A2235, Abclonal China) antibodies, anti-Collagen I (1:200; A22090, Abclonal China) antibodies, and anti-Collagen III (1:100; db14567, diagbio China) antibodies after antigen retrieval and nonspecific blocking (Maxin, KIT-7710, China). Following overnight primary antibody incubation at 4 °C and 30 min secondary antibody treatment at room temperature, immunoreactivity was visualized with DAB and counterstained with hematoxylin. Masson’s trichrome staining was performed using the above sections as follows. After deparaffinization, the sections were stained with Masson’s Trichrome Stain Kit (Solarbio, G1340, China), followed by neutral balsam sealing. Observations were performed using microscope (Olympus, Japan), and semi-quantitative analysis was performed using the ImageJ software.

### 4.11 Fluorescence staining of ferrous ions in living cells

AML12 cells were stimulated as described above, then HBSS (Servicebio, G4203, China) containing 1 μmol/L FerroOrange (Dojindo, F374 Japan) was added and incubated for 30 min at 37 °C in a 5% CO_2_ incubator. The cells were observed under an Olympus fluorescence microscope (Japan).

### 4.12 Statistical analysis

Statistical analyses were performed using Prism10 (GraphPad, USA), and all values are expressed as mean ± SD. In this study, the T-test was employed to compare data between two groups, while a two-way ANOVA was utilized for comparisons among multiple groups. The boxes and error bars in the figures indicate the mean value and standard deviation, respectively. Statistical significance was detected if *p* < 0.05.

## Supporting information

S1 Table Primary antibody dilution ratio and manufacturer in this study. (DOCX)

S1 Fig Grouping and treatment of mice. (DOCX)

S2 Fig *C. sinensis* ESPs blocked GSH synthesis in AML12. (DOCX)

S3 Fig *C. sinensis* ESPs induced AML12 ferroptosis by inhibiting Nrf2. (DOCX)

S4 Fig Inhibition of ferroptosis inhibits HSCs activation by *C. sinensis* ESPs. (DOCX)

S5 Fig Fe^2^+ accumulation promoted ferroptosis by regulating HO-1 and TFRC in advanced stages of clonorchiosis. (DOCX)

S6 Fig Fer-1 exhibited no toxic effects on the adults of *C. sinensis.* (DOCX)

S1 Data Excel spreadsheet containing, in separate sheets, the underlying numerical data and statistical analysis for Figure panels in the main text. (XLSX)
