# Supplementary material for: Hepatic ferroptosis induced by Clonorchis sinensis exacerbates liver fibrosis
Source: PLoS Negl Trop Dis. 2025 Jun 2;19(6):e0013164. doi: 10.1371/journal.pntd.0013164 (PMC12151476; doi:10.1371/journal.pntd.0013164)
Supplement: S1 Table — (DOCX) [file pntd.0013164.s001.docx]

#### S1 Table. Primary antibody dilution ratio and manufacturer

| Primary antibody | Dilution ratio | Manufacturer |
| --- | --- | --- |
| Rabbit anti-GPX4 monoclonal antibody | 1:1000 | Selleck,USA |
| Rabbit anti-Nrf2 polyclonal antibody | 1:1000 | Abclonal,China |
| Rabbit anti-SLC7A11 monoclonal antibody | 1:1000 | Abcam,USA |
| Rabbit anti-Smad2/3 polyclonal antibody | 1:1000 | Abcam,USA |
| Rabbit anti-phosphorylated Smad2/3 polyclonal antibody | 1:1000 | Abcam,USA |
| Mouse anti-GAPDH monoclonal antibody | 1:10000 | Proteintech,China |
