# Supplementary figures and images for: Hepatic ferroptosis induced by Clonorchis sinensis exacerbates liver fibrosis
Source: PLoS Negl Trop Dis. 2025 Jun 2;19(6):e0013164. doi: 10.1371/journal.pntd.0013164 (PMC12151476; doi:10.1371/journal.pntd.0013164)

**S1 Fig Grouping and treatment of mice**


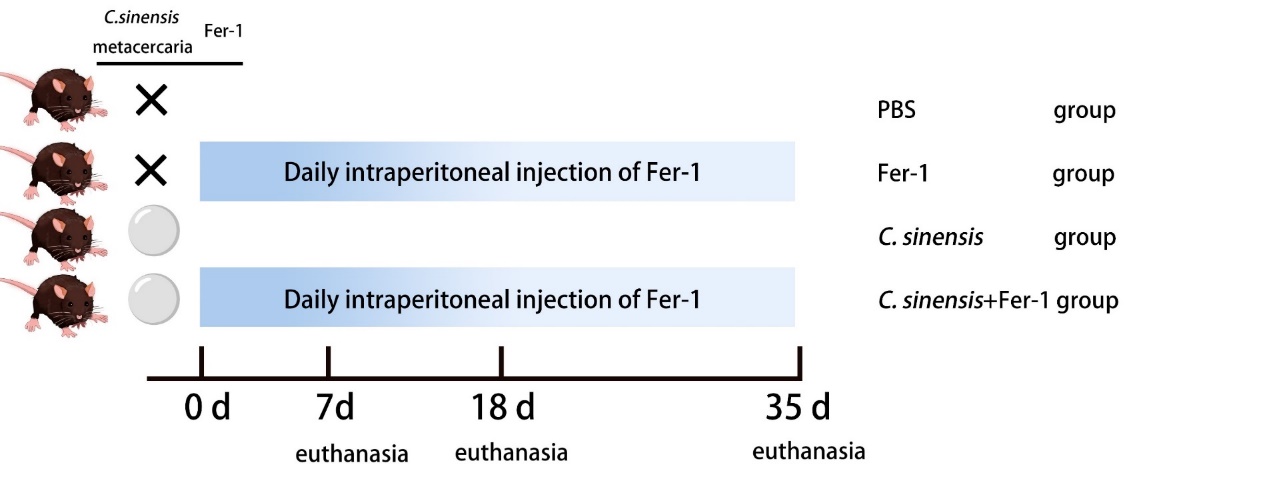


Mice were divided as depicted in the picture

Supplement: S1 Fig — (DOCX) [file pntd.0013164.s002.docx]
