# Supplementary material for: Hepatic ferroptosis induced by Clonorchis sinensis exacerbates liver fibrosis
Source: PLoS Negl Trop Dis. 2025 Jun 2;19(6):e0013164. doi: 10.1371/journal.pntd.0013164 (PMC12151476; doi:10.1371/journal.pntd.0013164)
Supplement: S2 Fig — (DOCX) [file pntd.0013164.s003.docx]

**S2 Fig** ***C. sinensis* ESPs blocked GSH synthesis in AML12**


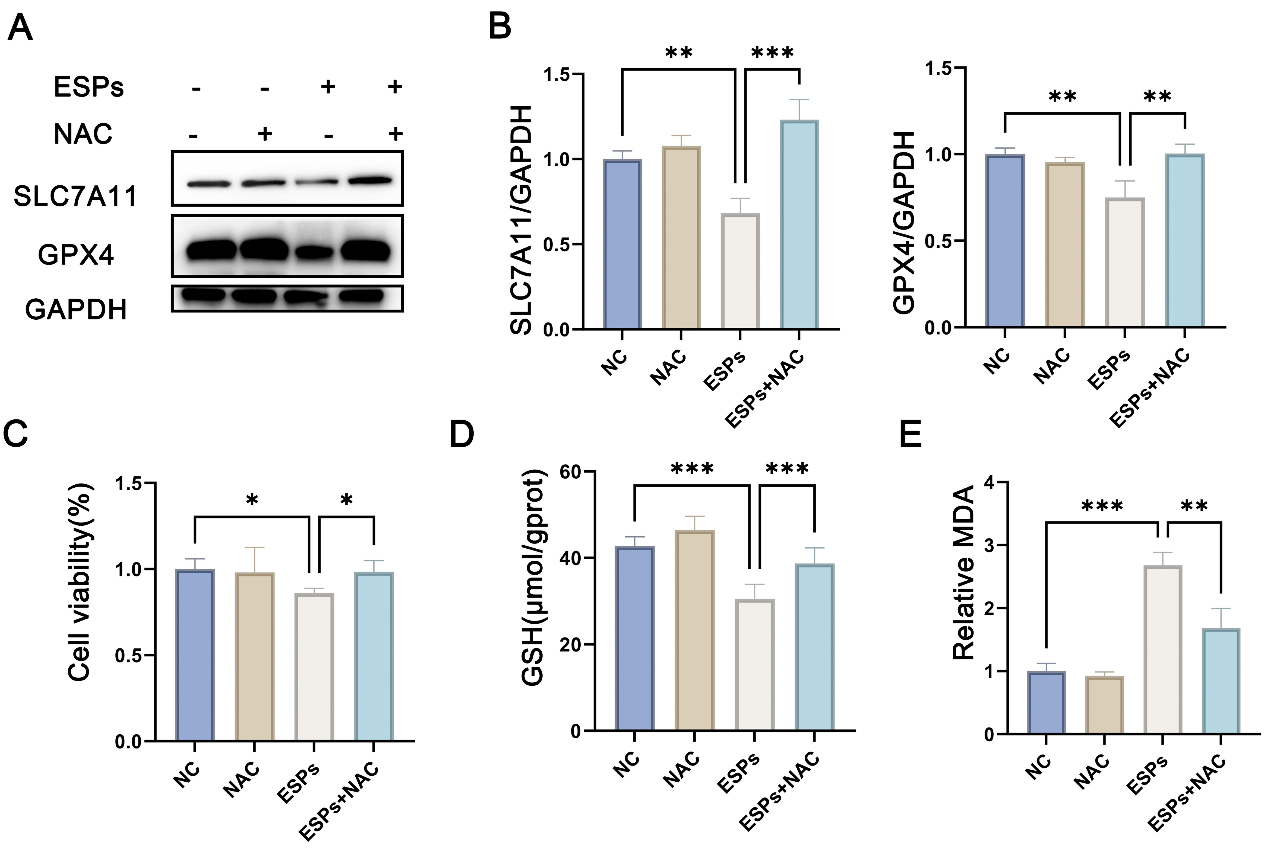


**S2 Fig *C. sinensis* ESPs blocked GSH synthesis in AML12.** AML12 cells were pretreated with NAC (1 mM) for 1 h, and then stimulated with ESPs (100 μg/mL) for 24 h. (A) SLC7A11, GPX4 expression in AML12 were detected by western blot. (B) Relative gray values in (A) were analyzed by ImageJ software. (C) Cell viability was detected by CCK8 assay. (D) Reduced GSH content in AML12 was detected. (E) MDA content in AML12 was detected. Data are derived from at least three independent cell wells within one experiment; **p* < 0.05, ***p* < 0.01, ****p* < 0.001, ns means no significant difference.
