# Supplementary material for: Hepatic ferroptosis induced by Clonorchis sinensis exacerbates liver fibrosis
Source: PLoS Negl Trop Dis. 2025 Jun 2;19(6):e0013164. doi: 10.1371/journal.pntd.0013164 (PMC12151476; doi:10.1371/journal.pntd.0013164)
Supplement: S3 Fig — (DOCX) [file pntd.0013164.s004.docx]

**S3 Fig *C. sinensis* ESPs induced AML12 ferroptosis by inhibiting Nrf2**


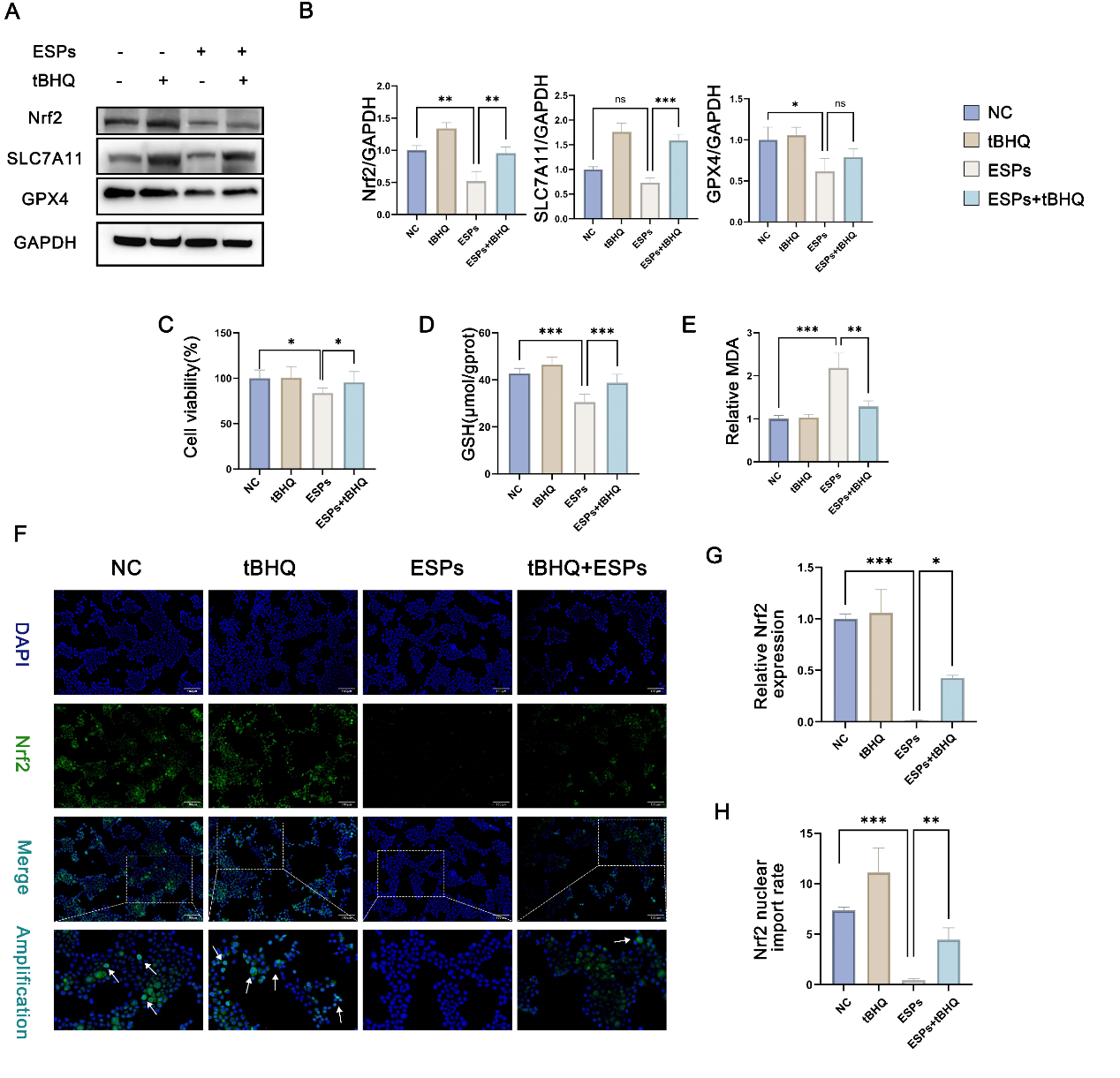


**S3 Fig *C. sinensis* ESPs induced AML12 ferroptosis by inhibiting Nrf2.** AML12 cells were pretreated with tBHQ (10 μM) for 1 h, and then stimulated with ESPs (100 μg/mL) for 24 h. (A) Nrf2, SLC7A11, GPX4 expression in AML12 were detected by western blot. (B) Relative gray values in (A) were analyzed by ImageJ software. (C) Cell viability was detected by CCK8 assay. (D) Reduced GSH content in AML12 was detected. (E) MDA content in AML12 was detected. (F) Nrf2 distribution and expression in cells were detected by immunofluorescence staining. Green fluorescence represented Nrf2, blue fluorescence represented nuclei, the white arrows points the nuclear translocation of Nrf2, scale bar = 100 μm. (G) Nrf2 expression was analyzed by ImageJ software. (H) Nrf2 nuclear import rate was analyzed by ImageJ software. Data are derived from at least three independent cell wells within one experiment; **p* < 0.05, ***p* < 0.01, ****p* < 0.001, ns means no significant difference.
