# Supplementary material for: Hepatic ferroptosis induced by Clonorchis sinensis exacerbates liver fibrosis
Source: PLoS Negl Trop Dis. 2025 Jun 2;19(6):e0013164. doi: 10.1371/journal.pntd.0013164 (PMC12151476; doi:10.1371/journal.pntd.0013164)
Supplement: S4 Fig — (DOCX) [file pntd.0013164.s005.docx]

**S4 Fig** **Inhibition of ferroptosis inhibits HSCs activation by *C. sinensis* ESPs**


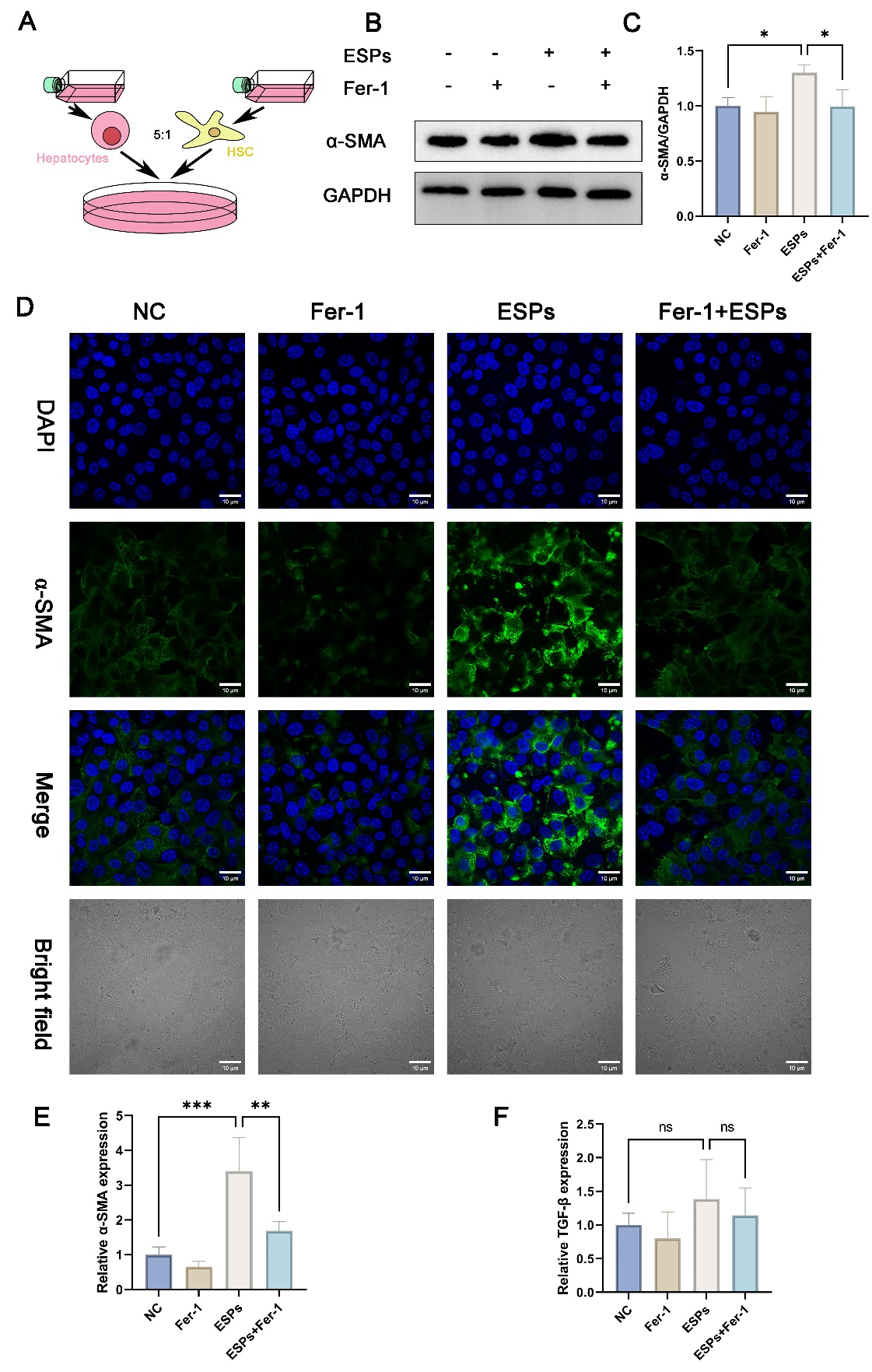


**S4 Fig Inhibition of ferroptosis inhibits HSCs activation by *C. sinensis* ESPs** (A) Hepatocytes and HSCs were digested using trypsin, counted, and co-cultured in a ratio of 5:1 hepatocytes to hepatic stellate cells, with a final cell density of 3×10^6^/mL. They were cultured in DMEM/F12 medium containing 1% FBS and 100 units/mL penicillin and streptomycin at 37°C, in a 5% CO2 environment. When the cells reached 70% confluence, experiments were performed. The serum-free medium was replaced 3 h in advance, and the cells were treated with Fer-1 (5μM) for 1 h. After treatment, ESPs were added for a 24 h co-incubation. The cells were then collected for the next steps of western blot and immunofluorescence experiments. (B) α-SMA expression in Co-culture system were detected by western blot. (C) Relative gray values in (A) were analyzed by ImageJ software. (D) Immunofluorescence for detection of cellular α-SMA expression, scale bar = 10μm. (E) Semi-quantitative analysis of α-SMA expression. (F) RT-qPCR was used to detect the expression level of TGF-β in AML12 cells. Data are derived from at least three independent cell wells within one experiment; **p* < 0.05, ***p* < 0.01, ****p* < 0.001, ns means no significant difference.
