# Supplementary material for: Hepatic ferroptosis induced by Clonorchis sinensis exacerbates liver fibrosis
Source: PLoS Negl Trop Dis. 2025 Jun 2;19(6):e0013164. doi: 10.1371/journal.pntd.0013164 (PMC12151476; doi:10.1371/journal.pntd.0013164)
Supplement: S5 Fig — (DOCX) [file pntd.0013164.s006.docx]

**S5** **Fig** **Fe^2+^ accumulation promoted ferroptosis by regulating HO-1 and TFRC in advanced stages of clonorchiosis.**


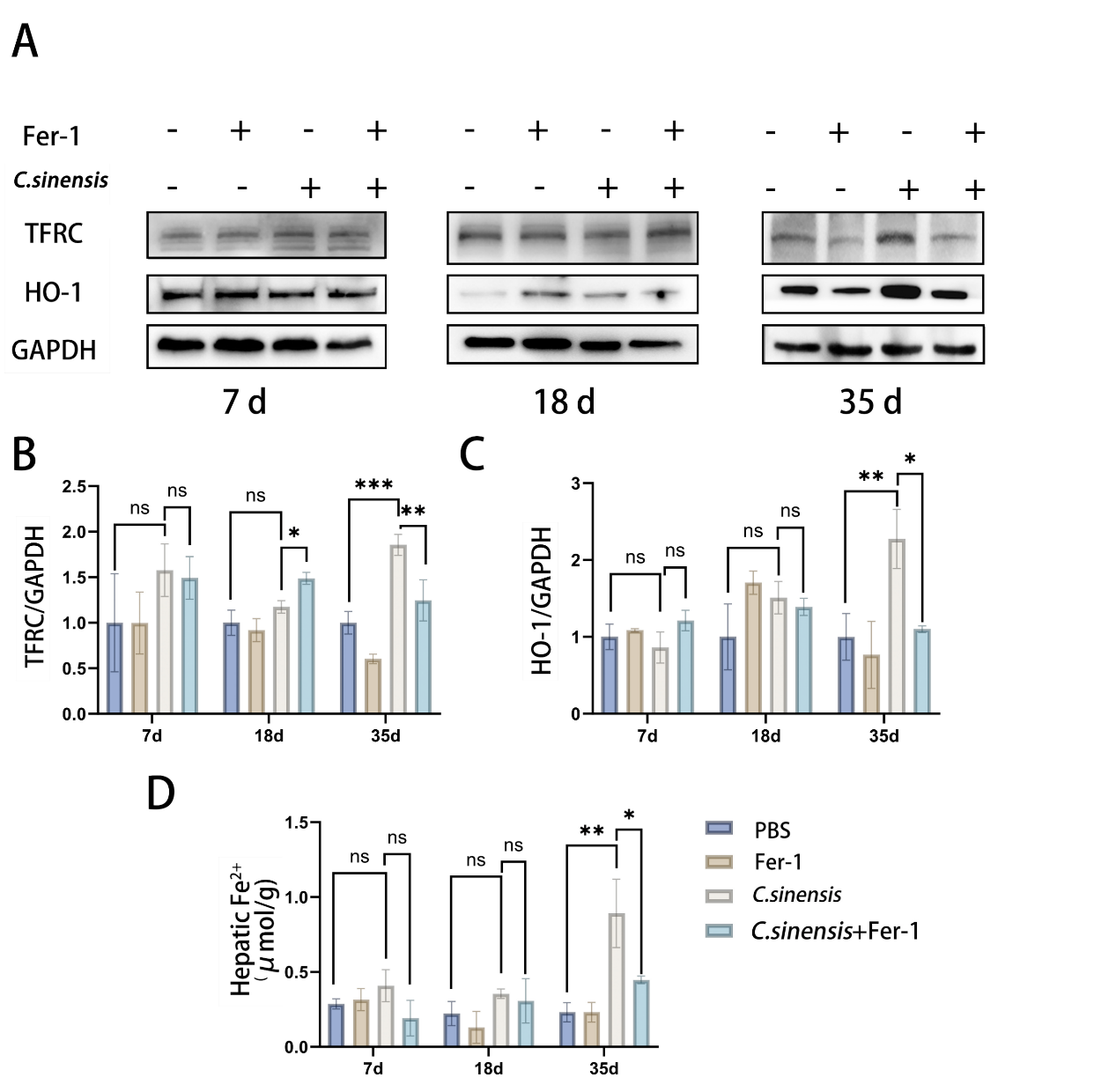


**S5 Fig Fe^2+^ accumulation promoted ferroptosis by regulating HO-1 and TFRC in advanced stages of clonorchiosis.** (A) TFRC, HO-1 expression in mice liver were detected by western blot. (B, C) Relative gray values in (A) were analyzed by ImageJ software. (D) Fe^2+^ content in mice liver was detected. Data are derived from at least three biologically independent mice per group; **p* < 0.05, ***p* < 0.01, ****p* < 0.001, ns means no significant difference.
