# Supplementary material for: Hepatic ferroptosis induced by Clonorchis sinensis exacerbates liver fibrosis
Source: PLoS Negl Trop Dis. 2025 Jun 2;19(6):e0013164. doi: 10.1371/journal.pntd.0013164 (PMC12151476; doi:10.1371/journal.pntd.0013164)
Supplement: S6 Fig — (DOCX) [file pntd.0013164.s007.docx]

**S6** **Fig Fer-1 exhibited no toxic effects on the adults of *C. sinensis*
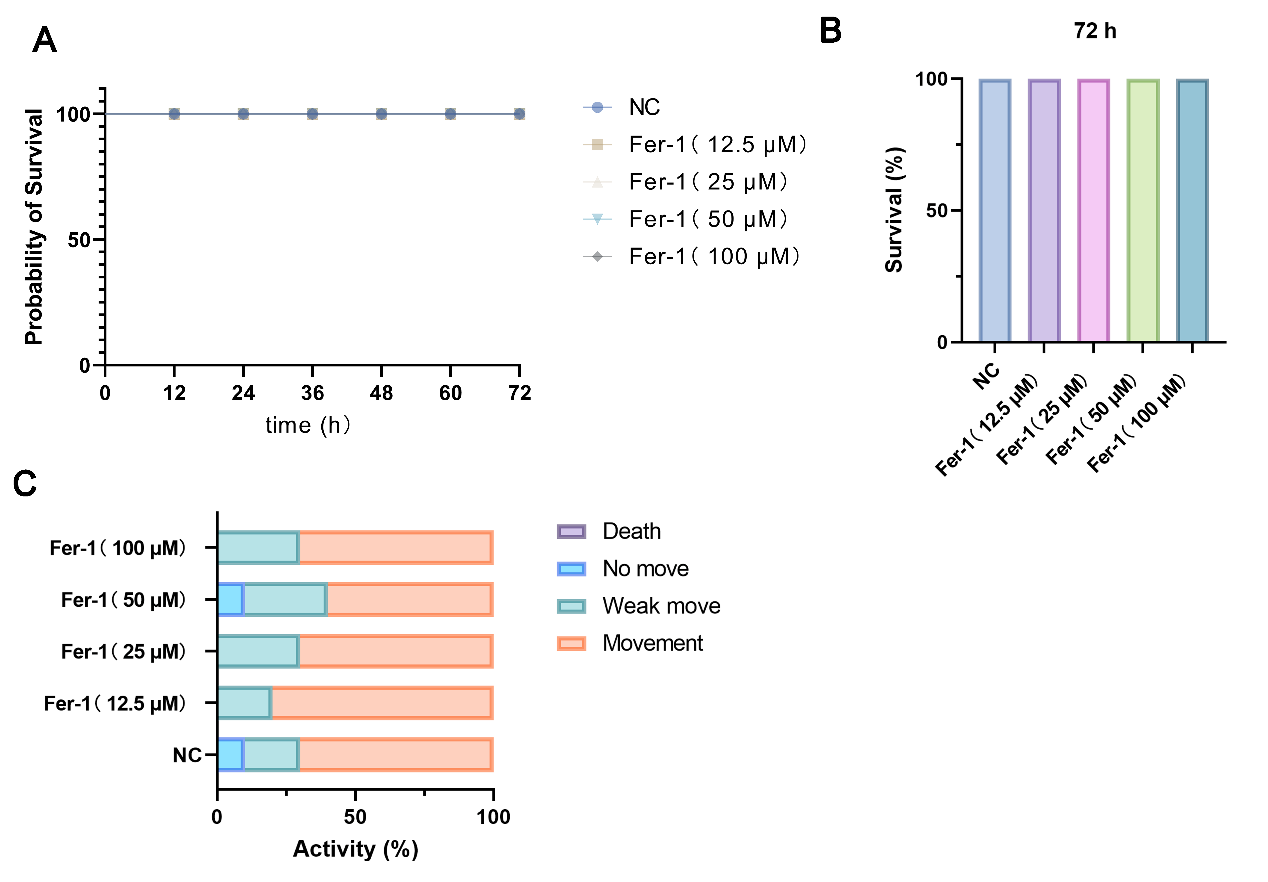
**

**S6 Fig Fer-1 exhibited no toxic effects on the adults of *C. sinensis.*** The adult *C. sinensis* were divided into five groups: negative control group, Fer-1 (12.5 μM), Fer-1 (25 μM), Fer-1 (50 μM), and Fer-1 (100 μM), with 10 adult worms in each group. They were placed in DMEM/F12 culture medium with the corresponding concentrations of Fer-1. After treatment, the survival rate was observed and recorded every 12 h. (A) Survival rate of *C. sinensis* under different concentrations of Fer-1 treatment. (B) Survival rate of *C. sinensis* under different concentrations of Fer-1 treatment at 72h. (C) Activity was based on *C. sinensis* adult motility, and these were quantified at 72h.
